# PREFERED SURGICAL TECHNIQUE USED BY ORTHOPEDISTS IN ACUTE ACROMIOCLAVICULAR DISLOCATION

**DOI:** 10.1590/1413-785220162405156380

**Published:** 2016

**Authors:** ALEXANDRE YUKIO NISHIMI, DEMETRIO SIMÃO ARBEX, DIOGO LUCAS CAMPOS MARTINS, CARLOS VINICIUS BUARQUE DE GUSMÃO, ROBERTO RANGEL BONGIOVANNI, LUCIANO PASCARELLI

**Affiliations:** 1. Hospital IFOR, Shoulder and Elbow Surgery Group, São Bernardo do Campo, SP, Brazil.; 2. Universidade Estadual de Campinas (Unicamp), Department of Orthopedics and Traumatology, Campinas, SP, Brazil.

**Keywords:** Acromioclavicular joint/surgery. Surgical procedures, operative. Orthopedica. Surveys and questionnaires.

## Abstract

**Objective:**

To determine whether training on shoulder and elbow surgery influences the orthopedist surgeons' preferred technique to address acute acromioclavicular joint dislocation (ACD).

**Methods:**

A survey was conducted with shoulder and elbow specialists and general orthopedists on their preferred technique to address acute ACD.

**Results:**

Thirty specialists and forty-five general orthopedists joined the study. Most specialists preferred the endobutton technique, while most general orthopedists preferred the modified Phemister procedure for coracoclavicular ligament repair using anchors. We found no difference between specialists and general orthopedists in the number of tunnels used to repair the coracoclavicular ligament; preferred method for wire insertion through the clavicular tunnels; buried versus unburied Kirschner wire insertion for acromioclavicular temporary fixation; and time for its removal; and regarding the suture thread used for deltotrapezoidal fascia closure.

**Conclusion:**

Training on shoulder and elbow surgery influences the surgeons' preferred technique to address acute ACD. Level of Evidence V, Expert Opinion.

## INTRODUCTION

There are more than 60 procedures described for the surgical treatment of acute acromioclavicular dislocation (ACD). All techniques aim to restore joint congruence, which can be obtained via open or arthroscopic procedures, with anatomical or not anatomical reconstruction, and using various types of implants.^1^ ^-^ ^5^ To date, no technique proved superior to others from a clinical and radiological point of view.^1^ ^-^ ^3^ ^,^ ^5^ ^-^ ^9^ Therefore, the choice of technique to be used will depend on the surgeon's training, on the material available and personal preference.^10^

Basically, there are two populations of trained orthopedic surgeons that are able to surgically treat acute ACD: specialists in shoulder and elbow surgery, and general orthopedic surgeons. Specialization in shoulder and elbow aims to improve the technical and technological capabilities of the orthopedist. Thus, it is believed that specialization can modify the surgical and clinical reasoning of the orthopedists, influencing their decisions.

The objective of this study was to determine whether training in shoulder and elbow surgery influences the choice of the surgical technique for the management of acute ACD. To this end, a questionnaire was applied to orthopedic specialists and general orthopedic surgeons, in order to determine the technical differences between these two groups of surgeons. 

## MATERIALS AND METHODS

The study was submitted for assessment by the institutional Research Ethics Committee and approved under protocol number 1627521. We interviewed orthopedists with and without specialization in shoulder and elbow surgery. Each orthopedist responded a questionnaire on the technique used and further details for the surgical treatment of acute ACD. (Annex 1)

Only orthopedists holding a title of specialist - Orthopedics and Traumatology Specialist (OTS) recognized by the Brazilian Society of Orthopedics and Traumatology (*Departamento de Ortopedia e Traumatologia (DOT/FMUSP)*, SBOT) that have operated at least one acute ACD in the previous year were included in the sample.

Orthopedists specialist (OTS) was defined as professionals with titles recognized by SBOT who have completed an internship in shoulder and elbow surgery accredited by the Brazilian Society of Shoulder and Elbow Surgery (*Sociedade Brasileira de Cirurgia do Ombro e Cotovelo*, SBCOC).

Statistical analysis was performed with SPSS 17.0 software, which calculated absolute and relative frequencies for qualitative variables. We used the chi-square test or Fisher's exact test to assess the homogeneity between proportions. Was adopted α = 0.05 as significance level.

## RESULTS

The questionnaire was applied to 75 orthopedists which matched the inclusion criteria, 30 specialists (40%) and 45 generalists (60%).

Most generalists operated up to five ACD the previous year; while most specialists operated more than five ACD specialists the previous year, and they integrate the group with the largest number of orthopedic surgeons who operated more than 10 ACD cases in that period. (Table 1)

**Table 1:**
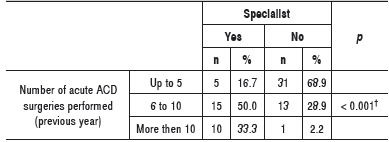
Absolute and relative frequency of surgery for acute ACD performed the previous year between specialists and non-specialists. ^†^Descriptive level of probability of the chi-square test.

There were no differences between groups regarding OTS-SBOT time , in years, of the OTS-SBOT time was equal to shoulder and elbow surgery time for the specialists group. (Table 2)

**Table 2:**
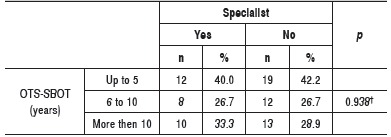
Absolute and relative frequency of the time of acquisition of the Orthopedics and Traumatology Specialist title (OTS-SBOT) among specialists and non-specialists. ^†^Descriptive level of probability of the chi-square test.

The most used technique among specialists and generalists was suture-anchor modified Phemister's^11^ technique or wiring to repair coracoclavicular ligaments.^12^ Of the modifications of Phemister's technique, the most used among generalists was the one with anchors; and the most used among specialists was wiring. Considering the technics alone (separating Phemister's changes), the most used among the specialists was the endobutton technique. On the contrary, no non-specialist orthopedist used the endobutton technique on acute ACD surgery. (Table 3)

**Table 3:**
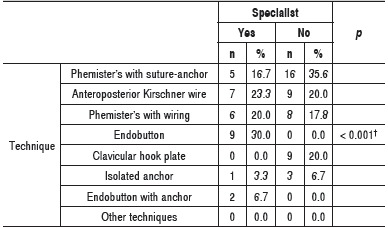
Absolute and relative frequency of the preferred surgical technique among specialist and non-specialist surgeons. ^†^Descriptive level of probability of the exact Fisher's test.

Both specialists and generalists who used Phemister's modified techniques, or coracoclavicular repair technique and anterior to posterior temporary fixation with Kirschner wire toward the scapula,^13^ mostly used two clavicular tunnels to repair coracoclavicular ligaments. (Table 4) There was no difference between the proportion of specialists and generalists regarding the number of tunnels used.

**Table 4:**
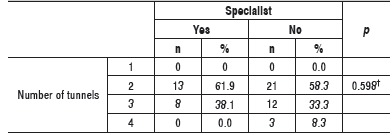
Absolute and relative frequency of the number of tunnels used to repair coracoclavicular ligaments of Phemister's modified technique. ^†^Descriptive level of probability of the exact Fisher's test.

In cases where the acromioclavicular joint was temporarily fixated with intramedullary Kirschner wire (Phemister's technique and its modifications) or anteroposterior toward the scapula,^13^ no difference regarding burying or not the Kirschner wire into the patient's subcutaneous tissue was observed between specialists and generalist, as well as the time of permanence of Kirschner wire. (Table 5) However, most surgeons, specialists or generalists, preferred to remove the Kirschner wire after six to eight weeks.

**Table 5:**
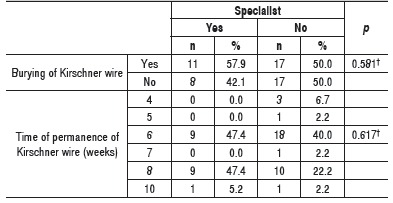
Absolute and relative frequencies of the number of surgeons who buried and did not bury the Kirschner wire under the cutaneous tissue in cases of temporary fixation of the acromioclavicular joint and permanence of the Kirschner wire and the number of surgeons who buried and did not bury the Kirschner wire under the cutaneous tissue in cases of temporary fixation of the acromioclavicular joint. Descriptive level of probability of the chi-square test.

Half of the specialists used to pass the repair of coracoclavicular ligaments through the clavicle tunnel using a Ethibond(tm) thread needle (Ethicon Inc, NJ, USA); while the other half passed it through Aciflex(tm) wire (Ethicon Inc, NJ, USA). This wire was hardly used by generalists, which mostly used Ethibond(tm) wire needle. Only one non-specialist used cerclage wire, and one non-specialist used nylon wire. (Table 4) For closing the deltotrapezoidal fascia, Vicryl(tm) thread (Ethicon Inc - NJ, USA) was preferred by most specialist or not specialist surgeons, followed by Ethibond(tm) wire. A minority of specialists used wire Fiber Wire^(r)^ (Arthrex, Inc. - FL, USA), and no one used nylon; while the minority of generalists used nylon wire, but no one used Fiber Wire^(r)^. (Table 6)

**Table 6:**
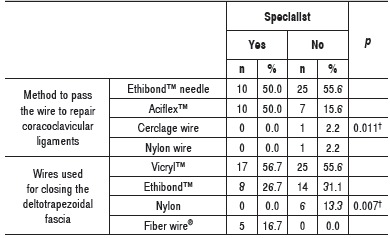
Absolute and relative frequencies of the method used to pass the wire to repair the coracoclavicular ligaments through the clavicular tunnel and wire used for closing the deltotrapezoidal fascia. ^†^Descriptive level of probability Fisher's exact test.

## DISCUSSION

Surgical treatment of acute ACD can be done in different ways, with similar clinical outcomes.^1^ ^-^ ^3^ ^,^ ^5^ ^,^ ^7^ ^-^ ^9^ As the specialization in shoulder surgery and elbow internship aims to improve the technical and technological capabilities of the orthopedic surgeon, it has been questioned whether this specialization influences the preference for the technique used on surgical treatment of acute ACD.

In this study, the time of Orthopedists and Traumatology Specialists (OTS) according to the criteria of the Brazilian Society of Orthopedics and Traumatology (SBOT) and generalist were similar, which reduces the influence of surgical experience and improves the correlation of the findings with the academic background. Despite the similarity, specialists operated more acute ACD cases the previous year, suggesting that these cases were selected to be treated by specialists.

No non-specialist orthopedic surgeon used the endobutton technique, and some were even unfamiliar with it (data not shown). On the contrary, no specialists used hook clavicular plate, while this practice is adopted by 20% of generalists. Despite the fact that endobutton may cause an inflammatory reaction,^14^ the non-use of this plate by specialists may be motivated by the recognized need for its removal when it causes discomfort to the patient.^3^ Temporary fixation of anterior to posterior Kirschner wire toward the scapula was adopted by 23.3% of specialists and 20% of generalists. This is not an established technique as Phemister's, but it has been described by surgeons in charge of specialized services on our midst.^13^ These findings reinforce the influence of academic training in surgical preference.

In accordance with this fact, some technical details were preferred by specialists as compared to generalists, as the largest proportion of Aciflex(tm) users to pass the repair of coracoclavicular ligaments through the clavicle tunnels, and Fiber Wire^(r)^ for closing the deltotrapezoidal fascia; whereas, among the generalists, there was a greater proportion of cerclage wire or nylon users to repair coracoclavicular ligaments through clavicle tunnels, and nylon wire for closing the deltotrapezoidal fascia. The higher cost of Aciflex(tm) and Fiber Wire^(r)^ inhibits their use mainly in public hospitals, where most of the training of specialists and generalists orthopedists is done. It is believed that specialists have more contact with these technologies and suppliers of these materials, justifying their increased use in acute ACD surgeries.

Despite the observed differences, most specialists used the same amount of tunnels to repair coracoclavicular ligaments as generalists. Most individuals in both groups also used the same passage technique to repair such ligaments. The rationale for conducting two transclavicular tunnels is given by the existence of two coracoclavicular ligaments; however, no studies were found comparing the use of one to four tunnels for the same technique.^15^ ^,^ ^16^

There was also consensus on: the wire used for closing the deltotrapezoidal fascia; the length of Kirschner permanence; and the choice of burying or not the Kirschner wire into the subcutaneous tissue. These preferences were justified by the known time of six to eight weeks for stabilization of the acromioclavicular joint and concerns regarding postoperative infection of the Kirschner wire orifice.^17^ ^,^ ^18^

Interestingly, although there are studies showing similar clinical and radiological results as to open techniques, not even specialists used minimally invasive or arthroscopic techniques.^3^ ^,^ ^4^ ^,^ ^10^ ^,^ ^19^ ^,^ ^20^ The non-availability of suitable material at the hospital, lack of training during specialization, long learning curve and increased surgical time may justify the non-use of these techniques. However, some technical details were not influenced by specialization. Moreover, even with specialization, shoulder and elbow surgeons do not perform minimally invasive or arthroscopic surgery. Further research may lead to a better understanding of the reasons, as well as regarding the correlation of surgical preference with clinical and radiographic results.

## CONCLUSION

Based on the findings of this study, we concluded that specialization in shoulder and elbow surgery changes the orthopedists' preference on the technique used in the surgical treatment of acute ACD. 
